# Exploring the antecedents of customers’ willingness to use service robots in restaurants

**DOI:** 10.1007/s11628-022-00509-5

**Published:** 2022-11-02

**Authors:** Sebastian Molinillo, Francisco Rejón-Guardia, Rafael Anaya-Sánchez

**Affiliations:** Department of Business Management, University Institute of Tourist Investigation, Intelligence and Innovation, University of Malaga, 29010 Malaga, Spain

**Keywords:** Service robots, Willingness to accept, Intention to recommend, Objections to use, Interaction quality perception, Perceived safety, Anthropomorphism

## Abstract

This study examines the willingness of customers to accept, and their intention to recommend, the services provided by service robots in restaurants. A mixed-methods research approach was taken to evaluate a theoretical model based on behavioural reasoning theory (BRT). The results demonstrated the important influence of positive attitudes and objections to the use of service robots on consumers’ willingness to use service robots, as well as their intention to recommend restaurants that use them. Among the main aspects that affect attitudes and objections, we found hedonic perceptions, perceived safety, interaction quality perception and anthropomorphism.

**Supplementary Information:**

The online version contains supplementary material available at 10.1007/s11628-022-00509-5.

## Introduction

To date, humans have been the service providers par excellence, but with the advent of advanced digital technologies (e.g. artificial intelligence—AI, and the internet of things—IoT), it is increasingly common to see intelligent robots employed to provide support services or to replace workers in various functions (Belanche et al. 2021a; Huang and Rust 2018; Wirtz et al. 2018). Service robots are mechanical devices that mimic human behaviours to, autonomously or semi-autonomously, provide services (Chiang and Trimi 2020). These robots are being successfully employed in various sectors, such as hospitality, restaurants, airports and retailing (Belanche et al. 2021c; Chiang and Trimi 2020), where they are more capable than humans in certain areas or are used to carry out dangerous or unpleasant tasks. In addition, in the recent COVID-19 situation, service robots proved their usefulness in a wide range of services and industries where social distancing was important (Flavián and Casaló 2021; Matthews 2020).

Nonetheless, there is still a gap between the levels of service provided by robots and by humans, such that researchers and customers harbour some doubts about their use (Arici et al. 2022; Prentice et al. 2020). Specifically, not all consumers are positively disposed to accept robots, citing negative aspects such as poorer quality service, lack of human contact and ethical concerns (Huang and Rust 2018). Moreover, the negative perceptions generated by service robots have been shown in some cases to exceed the positive perceptions they evoke, thereby harming the overall service experience (McLeay et al. 2021).

Most research into service robots has focussed on their design and development, and few empirical studies have examined the quality of service they provide (Chiang and Trimi 2020). Similarly, few works have examined positive customer behaviours, such as their intention to recommend establishments that use robots, or their propensity to use service robots (Lin et al. 2020; Yoganathan et al. 2021). In this sense, previous studies have explored attitudes and behaviours in the context of the employment of AI in service delivery (Belanche et al. 2020a), mainly, as noted by Gursoy et al. (2019), based on traditional technology acceptance theories (e.g. Lu et al. 2019; Zhong et al. 2020). Essentially, these models incorporated users’ cognitive beliefs, such as perceived usefulness and perceived ease of use, but few evaluated their perceptions of the quality of their interactions with the service provider (Choi et al. 2020). In addition, service robots often feature levels of anthropomorphism that may invalidate the use of classic theoretical frameworks of technological acceptance (Lin et al. 2020; Yang et al. 2021).

Therefore, in recent times, researchers have begun to use other theories to explain how relationships develop between service robots and consumers, and to identify the reasons why consumers use these technologies and their underlying motivations for so doing (Web Appendix A). For example, Fan et al. (2020) used Wirtz et al.’s (2018) service robot acceptance model in their conceptual work on the employment of service robots on the frontline. Gursoy et al. (2019), based on cognitive appraisal theory and cognitive dissonance theory, proposed a theoretical model of AI device use acceptance (AIDUA), which was also applied by Lin et al. (2020). Lu et al. (2021) used appraisal theory, Choi et al. (2020) used the computers are social actors paradigm, while Belanche et al. (2020c, 2021c) used the attribution theory framework.

The present study analyses, based on behavioural reasoning theory (BRT), consumers’ intentions to use service robots in restaurant services. BRT proposes that the reasons for, and against, behaviours are the fundamental antecedents of overall motives (e.g. attitude) and consumer intentions (Westaby 2005). In the BRT, reasons are contextualised to the specific behaviour under investigation, and are defined as "specific cognitions connected to a behavioural explanation" (Westaby 2005, p. 100). Previous studies have demonstrated the usefulness of BRT in explaining the acceptance of technological innovations such as mobile shopping (Gupta and Arora 2017), artificial intelligence (Gesk and Leyer 2022), augmented reality (Manchanda and Deb 2021) and chatbots (Lin et al. 2022).

The main objective of this study is to explain customers’ predispositions to accept the service robots that are used to complement human work in the hospitality sector (Belanche et al. 2021c; Lu et al. 2019). To this end, a model, based on the BRT, is proposed that explores the effects of consumers’ reasons for using service robots on their willingness to accept the devices and intention to recommend restaurants that employ them. A two-stage study was carried out. First, a qualitative study identified the context-specific reasons that consumers highlight when evaluating a restaurant that uses service robots. Five specific aspects were identified: anthropomorphism, hedonic perceptions, interaction quality perceptions, safety perceptions and objections. Second, a theoretical relationship model, based on the BRT, was designed, and validated using quantitative data, to explain consumers’ intentions to recommend establishments operating service robots and their willingness to accept them in restaurants.

## Theoretical framework and hypotheses development

### Behavioural reasoning theory

BRT was formulated by Westaby (2005) to try to explain the determinants of consumers’ intentions and behaviours. Related to traditional theories such as the theory of reasoned action (TRA) (Fishbein and Ajzen 1975), and its subsequent evolution, the theory of planned behaviour (TPB) (Ajzen 1991), the BRT is characterised by its strong emphasis on the argument that context-specific factors are determinants of behaviours. The TRA and the TPB propose that attitudes towards behaviours, subjective norms and perceived behavioural control are determinants of consumer intentions, and put less focus on belief concepts (e.g. behavioural beliefs, normative beliefs, control beliefs) as antecedents. Instead, Westaby stated that "reasons serve as important linkages between people's beliefs, global motives (e.g. attitudes, subjective norms and perceived control), intentions and behaviour" (Westaby 2005, p. 98). Reasons are subjective factors that people use to adopt and maintain behaviours (Gupta and Arora 2017). Reasons are not general, they depend on specific contexts (e.g. the use of service robots in restaurants), and are classified in two categories, "reasons for" and "reasons against" (Lalicic and Weismayer 2021).

Recent studies have proven that BRT is valid for explaining consumer technology adoption behaviour (e.g. Gesk and Leyer 2022; Lalicic and Weismayer 2021; Lin et al. 2022). In the present study, BRT is used to develop an understanding of how the factors that argue for, and against, the use of service robots explain consumers' intentions towards the use of service robots in restaurants.

### Anthropomorphism

Anthropomorphism “involves the attribution of human characteristics, human form, and human behaviour to something that is nonhuman, such as a robot or a computer” (Jang and Lee 2020, p. 3). In recent years, studies have demonstrated the positive effects of anthropomorphism on users’ perceptions of AI-powered robots and devices (e.g. Belanche et al. 2021b). Their designers argue that customers are more willing to use AI-powered service agents with a high degree of anthropomorphism, which they say enhances their implementation and utilisation (Qiu et al. 2020). The anthropomorphisation of a product or service can lead, based on the individual's perceptions, to positive feelings or affect (Aggarwal and McGill 2007). Anthropomorphism has a positive effect on the evaluation of AI-powered assistants (Li and Sung 2021) and is positively associated with attitudes towards the use of AI travel advisors (Martin et al. 2020) and robot concierges (Shin and Jeong 2020). Anthropomorphic AI-powered assistants evoke lower psychological reactance than non-anthropomorphic assistants (Pizzi et al. 2021). In addition, in the context of AR-mediated m-commerce, it has been shown that anthropomorphised technologies generate positive effects (attitudes) and reduce negative effects and objections (Manchanda and Deb 2021). Based on these arguments, we propose the following hypotheses:

**H1**: Anthropomorphism has a negative effect on objections to the use of service robots.

**H2**: Anthropomorphism has a positive effect on attitudes towards the use of service robots.

### Hedonic perceptions

Hedonic perceptions about AI relate to the mental image that customers have of the pleasure or fun they expect to experience when using AI devices in service provision (Gursoy et al. 2019; Lu et al. 2019). These thoughts may play an important role in the determination of consumers’ acceptance behaviours towards certain technologies, given they are key drivers of decision-making (Wei et al. 2016).

Some studies have argued that the hedonic aspects of service robots are determinant in their integration into service delivery systems (Lu et al. 2019), and that their perceived value is directly conditioned by the consumer’s hedonic perceptions (Čaić et al. 2020). When users perceive that using service robots is fun and pleasant their perceptions of the benefits that they can obtain from using them will increase and, in addition, their perceptions of the effort or difficulty involved in using them will decrease (Lin et al. 2020). It has also been shown that consumers with hedonic motivations for using AI-powered devices are likely to have positive attitudes towards using them (Gursoy et al. 2019), and that hedonic motivations are among the factors that exert the greatest positive effects on intention to adopt this type of device (Vimalkumar et al. 2021). Based on the above points, we propose the following hypotheses:

**H3**: Hedonic perceptions have a negative effect on objections to the use of service robots.

**H4**: Hedonic perceptions have a positive effect on attitudes towards the use of service robots.

### Perceived safety

Perceived safety describes both the user's perception of the level of danger (s)he might face when interacting with a robot, and the level of comfort (s)he might experience during the interaction (Bartneck et al. 2009). It is logical to conclude that perceived safety is a key issue for humans in their interactions with robots, and the concept has attracted considerable attention in the literature, where it has been demonstrated that perceptions of risk, insecurity and safety influence intentions to use AI-powered devices and service robots (e.g. Flavián et al. 2021). Thus, the perceived safety of service robots is expected to affect the perceived value of the service, particularly in terms of interaction quality (Bartneck et al. 2009; Kleijnen et al. 2007). In consequence, the following hypothesis is proposed:

**H5**: Perceived safety has a positive effect on interaction quality perception.

Health-related risks are currently particularly relevant in the context of AI-powered devices, given the global pandemic situation caused by COVID-19 (Lew 2020; Matthews 2020); the use of service robots positively impacts on perceived safety by reducing human interactions, and helps maintain the social distance recommended by health authorities for service provision (Kim et al. 2021), including in restaurant services (Chuah et al. 2022). In addition, interactions carry other perceived risks, such as concerns about privacy and the security of personal data, which affect attitudes towards AI-powered devices (McLean and Osei-Frimpong 2019). Thus, the consumer’s perceptions of the safety of service robots will positively affect his/her attitudes towards their use (Jang and Lee 2020), and negatively affect his/her objections to their use. Based on the above definitions, and the relationships specified, the following hypotheses are proposed:

**H6**: Perceived safety has a negative effect on objections to the use of service robots.

**H7**: Perceived safety has a positive effect on attitudes towards the use of service robots.

### Interaction quality perception

Interaction quality is the consumer’s perception of the degree of quality of the process and provider–customer interaction during service delivery (Choi et al. 2020). It has been argued that, in the hospitality sector, the quality of the interaction between the customer and the service robot creates “a moment of truth” (Choi et al. 2020; Kandampully et al. 2018) and positively influences attitudes towards the service and the provider (e.g. Hwang and Ok 2013). Lee et al. (2021) argued that interaction quality is key to the success of AI-based services. This being the case, it is noteworthy that some authors consider that AI-powered devices are not, as yet, able to deliver the same level of interaction quality as that provided by human employees (Belanche et al. 2020c; Choi et al. 2020; Prentice et al. 2020). Robots’ lack of empathy and the personal touch, and their inability to deal with complex situations, make users reluctant to employ them in certain situations (Pelau et al. 2021). However, Choi et al. (2020) argued that the capabilities of, and quality of service (including quality of interaction) delivered by, robots are improving. Enhancing the quality of AI–human interaction is of great importance for the incorporation of AI into services (Bock et al. 2020). We believe that where users form good interaction quality perceptions, their attitude towards service robots will improve and objections to using them will reduce. Therefore, the following hypotheses are proposed:

**H8**: Interaction quality perception has a negative effect on consumers’ objections to the use of service robots.

**H9**: Interaction quality perception has a positive effect on consumers’ attitudes towards the use of service robots.

### Objections to the use of service robots

Just as the reasons users have for employing new technologies positively influence their attitudes towards their use, objections to adoption exert a negative influence (Claudy et al. 2015). Consumers form their attitudes towards a given behaviour based on their assessment of the reasons for, and against, undertaking the behaviour, and use this assessment to justify the behavioural choices they make (Westaby 2005). Consumers’ objections to the use of a technology, that is, their strong reasons against using it, are part of a cognition process that includes attitude (Anderson and Pirolli 1984). Previous studies have shown that the reasons users have for not wanting to adopt new technologies have a negative effect on their attitudes towards these smart technologies as virtual agents (Lalicic and Weismayer 2021), chatbots (Lin et al. 2022) and artificial intelligence devices (Gesk and Leyer 2022). Consequently, the following hypothesis is proposed:

**H10:** Objections to use have a negative effect on attitudes towards the use of service robots.

Some technologies are rejected by their users as they threaten to replace people or dehumanise the service provision relationship (Lee and Lee 2020). Objections to the use of AI devices and robots reflect the individual’s rejection due, among other reasons, to his/her need for social contact (Chi et al. 2022; Gursoy et al. 2019). Previous studies have suggested that the consumer’s need for social interaction is one of the main challenges for the adoption of AI-powered service delivery devices, as his/her assessment of the level of employee–client social interaction is one of the determinants of perceived value. Thus, consumers might believe that services, especially tourist services, require human, empathetic, professional contact that cannot yet be offered by AI (Choi et al. 2020; Prentice et al. 2020). In addition, users may not want to adopt AI-powered devices due to their expectations of the effort they will have to expend to learn how to use them (Vimalkumar et al. 2021). Therefore, the following hypotheses are proposed:

**H11:** Objections to the use of service robots have a negative effect on intention to recommend them.

**H12:** Objections to the use of service robots have a negative effect on willingness to accept them.

### Attitudes towards service robots

Cognitive appraisal theory (Watson and Spence 2007) suggests that customers with positive attitudes towards AI devices will be more willing to accept their use during the service delivery process (Gursoy et al. 2019). Similarly, BRT proposes that attitudes towards adopting new technologies are a key antecedent of behavioural intentions (Westaby 2005). In this sense, several articles have shown that favourable attitudes towards AI-powered devices (e.g. voice assistants, chatbots) increased use (e.g. Belanche et al. 2019; Lalicic and Weismayer 2021) and recommendation intentions (e.g. Mishra et al. 2021). Shin and Jeong (2020) demonstrated that consumers’ intentions to adopt hotel concierge robots were positively influenced by their attitudes towards the devices. Chuah et al. (2022) showed that consumers’ attitudes towards the use of service robots in restaurants positively influenced willingness to use restaurants that employ robots. This response behaviour is a consequence of the attitudes that consumers develop through their information processing and their perceptions of the benefits they derive from using robots, which can mitigate their objections to use. Based on the above, the following hypotheses are proposed:

**H13:** Attitudes towards the use of service robots have a positive effect on intention to recommend them.

**H14:** Attitudes towards the use of service robots have a positive effect on willingness to accept them.

### Intention to recommend

Behavioural intentions have been defined as an asserted probability that the individual will engage in a certain behaviour (Oliver 2014). In the present study, intention to recommend is taken to be the stated probability that the individual will, in the future, recommend the services provided by AI-powered service robots to family, friends and other people (Ryu et al. 2008). The consumer’s willingness to accept the use of service robots refers to his/her acceptance of the use of AI-powered service robots in future service encounters (Chi et al. 2022; Gursoy et al. 2019). Therefore, it seems logical to conclude that if an individual intends to recommend a service, this intention will positively influence his/her willingness to accept that service. Therefore, the following hypothesis is proposed:

**H15:** Intention to recommend has a positive effect on willingness to accept service robots.

**Fig. 1:**
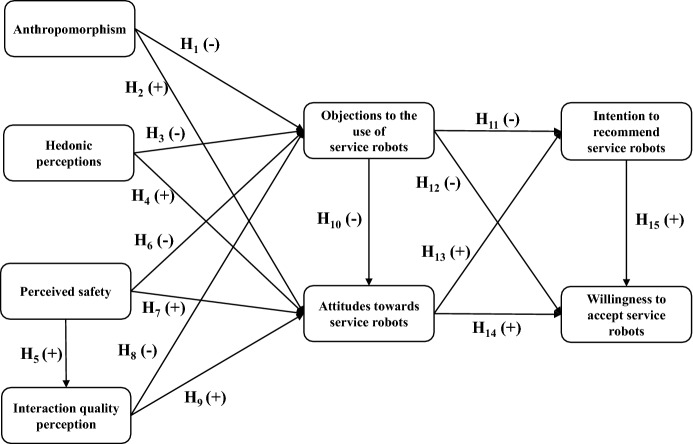
Model of willingness to accept service robot use

Figure 1 depicts the study model with its proposed relationships.

## Methodology

### Research overview

The present research employed a two-stage exploratory mixed-method approach: first, the gathering and analysis of qualitative data; second, the gathering of quantitative data (Creswell and Clark 2017). This type of design is associated with works where researchers want to explore topics rarely examined; based on qualitative data, hypotheses are proposed and a questionnaire is developed that is subsequently used to collect quantitative data with which to evaluate the hypotheses (Bell et al. 2019).

In this study, the use of an exploratory design is justified as customer responses to service robots are considered still an emerging phenomenon that needs to be explained through observation and subsequent confirmation or disconfirmation of proposed models (Schepers et al. 2022). Specifically, the two stages of the research were as follows: first, a content analysis of the TripAdvisor reviews of a restaurant that employs service robots (Study 1); and, second, a survey study that collected data from potential restaurant customers (Study 2). The content analysis allowed us to choose the constructs (reasons for, and against, the use of service robots in restaurants) that were incorporated, in the second stage, into the research model and the survey.

### Study 1: content analysis

The very many online reviews posted by users on platforms such as TripAdvisor and Google Maps are often analysed to investigate the consumer’s experience in tourism, hospitality and catering services (Meek et al. 2021). An online review is an unstructured text that can be used to collect data and gain insights into customers’ perceptions and experiences (Luo et al. 2021). Using online reviews to collect information has advantages, such as the easy availability of the data, the speed and simplicity of the collection process, and the researcher does not interfere in the process of the consumer’s expression of opinion (Lu and Stepchenkova 2015).

Taking these points into account, in the present study, the online reviews of a restaurant were analysed through content analysis to identify the reviewers’ main reasons for, and against, the use of service robots. Content analysis transforms qualitative information (i.e. review texts) into a quantitative description of the content expressed in a text. In a two-stage process, first, the different passages of the text are assigned into categories and, second, the frequencies at which the categories appear are analysed (Mayring 2014). Content analysis is a useful research tool for analysing a dataset to identify, categorise and describe, and to arrive at a systematic overview of, its content (White 2020).

In the present study, we used content analysis to explore comments posted on the review websites Google and TripAdvisor by customers of a restaurant in Madrid (Spain) which uses service robots to complement its waiter-based table service. The comments were collected based on days when the restaurant used robots, in the date range May 2019 to May 2020. The content analysis was carried out in three phases (Corbin and Strauss 1990): first, the comments were read to extract their meaning; second, through an analysis of each comment on the service provided by the robots; and third, manual, open, axial and selective coding. The categories were developed through a systematic inductive process following the steps recommended by Mayring (2014). First, the concept of each category was defined in the context of the research; then, texts of 25% of the reviews were analysed, line by line, to identify the different categories; thereafter, three expert researchers analysed all the reviews and coded all the material. To reduce the possibility of researcher subjectivity, Kassarjian’s (1977) recommendations were followed to ensure category reliability and inter-judge reliability (i.e. the percentage of agreement between judges analysing the same communications material). Thus, the reviews were coded and classified by three expert researchers. The researchers then compared the codings and reached a consensus where there was no precise agreement, and no reviews needed to be removed. In general, the percentage of agreement between the researcher peers (judges) was greater than 95%, so it is considered that there is no problem of reliability in this study.

The total number of reviews posted was 272, of which 158 made some reference to the service robots. In specific terms, the customers mainly mentioned aspects related to hedonic motivations (46.2% of the comments), anthropomorphism (35.6%) and outcome quality (27.9%); other aspects mentioned in less than 20% of the comments were objections to the use of IA devices, performance and effort expectancy, fear of the robots and perceptions of futurism. Of the comments made, 72.1% were positive, 21.2% negative and 3.9% neutral.

### Study 2: survey study

#### Research instrument

Data for the empirical evaluation of the model were collected through self-administered online surveys. The model’s structures were measured through reflective measurement scales validated in previous research and adapted to the context of service robots used in restaurants. Specifically, anthropomorphism was measured using five items adapted from Bartneck et al. (2009); hedonic perceptions were measured using three items adapted from Gursoy et al. (2019); interactive quality was measured using three items taken from Choi et al. (2020); perceived safety was evaluated using three items adapted from Jang and Lee (2020); attitude towards service robots was measured using four items adapted from Go and Sundar (2019); and objections to the use of service robots were evaluated using four items adapted from Gursoy et al. (2019). Regarding the dependent variables, intention to recommend was assessed using three items adapted from Jung et al. (2015), and willingness to accept service robots was measured through three items adapted from Gursoy et al. (2019). In all cases, 5-point Likert scales were used (1 = strongly disagree; 5 = strongly agree). As the original scales were in English, and the target population was Spanish, to maintain the accuracy of the original scales the questionnaire was translated into Spanish by a professional service. 

In addition, before the data collection stage, three experts (N.B. three other experts in the field, not the three coders) reviewed the questionnaire and made some small modifications to improve the understanding of the questions, while maintaining the meaning of the original scales. Subsequently, a test was carried out through convenience sampling, using 100 university students with knowledge of service robot-based interactions in restaurants. The Cronbach’s alphas of the measurement scales were all greater than 0.80 (Nunnally 1978).

Next, the data for the model assessment were collected. The respondents, prior to answering the questionnaire, randomly viewed one of two videos showing similar service robots operating in two restaurants (see Web Appendix B). Their viewing of the videos was monitored by means of a control check of their memories of the physical characteristics of the service robots.

Finally, 29 items were used to measure the 8 main constructs of the proposed model, and 6 sociodemographic questions were posed. No significant differences were observed in the responses of the participants based on the video they watched.

### Data collection

The target population was potential restaurant clients. The data were collected during July 2020 through a Spain-based online survey using a structured questionnaire. Convenience sampling was used, and to reduce possible bias, a link was posted on various Facebook pages and the survey was distributed through email lists. Before they began to answer the questionnaire, the participants were shown some text outlining the objectives of the research and explaining they needed to give their express consent to take part in the study. Their agreement was given by marking a voluntary participation consent box. After the participants provided their consent, they accessed the questionnaire through a link. First, a selection question was posed to exclude those outside the target population. Specifically, if they indicated they did not eat in restaurants, they were not allowed to answer the questionnaire. Thereafter, they viewed one of two videos which showed service robots operating in restaurants. They were asked to answer the questionnaire based on their opinions of the service robots they saw in the videos. Questions were then posed to provide data through which to measure the model and to gather information about the participants’ sociodemographic characteristics. Responses with repeat values were discarded.

### Data analysis procedure and sample

First, descriptive analyses were carried out using Jamovi, a free, open software based on the R statistical language (The Jamovi Project 2021). Despite its only recent development, Jamovi is used by researchers in a variety of disciplines, such as management (e.g. Adil 2020) and psychology (Besner et al. 2021). Table 1 depicts the sample’s characteristics. The final sample consisted of 645 potential customers with a mean age of 32.65 years (S.D. = 13.24), 58.5% being women. Some 37.3% of their families had four members, and 20.40% had three; 36.28% had completed university studies, and 25.89% secondary/high school studies; 45.58% were employees, and 33.03% were students; monthly family income was lower than €1801 for 51.59% of the participants.

**Table 1:** Sample characteristics

Demographics of sample			Number of observations			Percentage (%)
*Mean age, years (S.D.)*			32.65 (13.24)			
*Gender (n = 645)*						
Male			267			41.40
Female			378			58.60
*Family members (n = 645)*						
One			28			4.34
Two			118			18.30
Three			132			20.47
Four			241			37.36
Five or more			126			19.53
*Educational Level (n = 642)*						
Primary			17			2.65
Professional training			97			15.11
Secondary/High school			167			26.01
Higher Education/University			234			36.45
Postgraduates			127			19.78
*Occupation (n = 603)*						
Unemployed			31			5.14
Students			213			35.32
Employed			294			48.76
Self-employed			65			10.78
*Family Income (monthly) (n = 535)*						
Less than 1200			116			21.68
1201 to 1500€			82			15.33
1501 to 1800€			78			14.58
1801 to 2400€			102			19.07
2401 to 3000€			57			10.65
3000€ or more			100			18.69
*Previous experience with Service Robots (n = 625)*						
Once or none			367			58.72
Twice			99			15.84
Three times			77			12.32
Four times			35			5.60
Five or more			47			7.52
*Frequency of visits to restaurants (weekly)*						
Breakfast (mean; S.D.)			1.087 (1.502)			
Lunch (mean; S.D.)			1.206 (1.272)			
Dinner (mean; S.D.)			1.805 (1.271)			

Jamovi was also used to test for differences in the participants' responses based on the video they watched (video 1 vs video 2), through a Student’s t test of the mean values of the dependent variables "intention to recommend" and "willingness to accept service robots". No significant differences were found between the two groups of participants in the means of intention to recommend (M_video1_ = 3.290, SD_video1_ = 1.083; M_video2_ = 3.265, SD_video2_ = 1.019; t(644) = 0.292, p = 0.770) or the means of willingness to accept service robots (M_video1_ = 3.486, SD_video1_ = 1.084; M_video2_ = 3.290, SD_video2_ = 1.090; t(644) = -1.585, p = 0.113). Therefore, we can affirm that there were no differences between the participants based on the video they watched.

Subsequently, the model was quantitatively evaluated using partial least squares (PLS-SEM) with SmartPLS3 software (Ringle et al. 2015). PLS-SEM is considered an appropriate methodology to use with small samples, and when normality is not assumed (Hair et al. 2012). In the present study, it was not possible to guarantee that the data were distributed normally using the Shapiro–Wilk normality test or using the Kolmogorov–Smirnov test with JAMOVI software. In addition, the theoretical knowledge underpinning the relationships of the proposed model is still in development (Fornell and Bookstein 1982). The analysis was carried out in two stages: first, the reliability and validity of the constructs were verified, and then the stability of the estimates was verified through a bootstrapping procedure (5000 subsamples), with two-tailed tests, at a significance level of 0.05.

## Results

### Common method bias

As the data were collected from the same source through an identical collection method, common method bias (CMB) may be a problem (Podsakoff et al. 2003). First, a Harman's confirmatory factor analysis (CFA) single factor model test was conducted, followed by an unmeasured latent variable test (Markel and Frone 1998). If a single item has a total variance greater than 50%, it can introduce CMB into data and empirical conclusions (Podsakoff et al. 2003). In the present study, no single factor had a total variance over 30.52%, and the evaluation of all the factors introduced into the model explained 73.2% of the variance; this suggests that CMB should not be a significant problem for this dataset.

### Measurement model assessment

Table 2 shows the results of the evaluations of construct reliability and convergent validity. Five items (ANT5, HP2, ATR4, OU1 and OU4) were removed from the model as their factorial loads did not exceed 0.7. Following this modification, both the Cronbach's alpha (AC) and composite reliability (CR) values exceeded in all cases the minimum 0.8 suggested by Nunnally (1978). The average variance extracted (AVE) values exceeded the recommended minimum level of 0.5 (Fornell and Larcker 1981).

To verify discriminant validity, three valid PLS-SEM methods were followed: (i) the load coefficients must be greater than the cross-loads; (ii) the inter-construct correlations must be less than the square root of the AVEs (Table 3); (iii) the heterotrait-monotrait ratio of correlations (HTMT) must be less than 0.9 (Table 3). All values were below the recommended maximum thresholds. These results demonstrate the reliability and validity of the measures. Thus, the structural model is suitable for analysis.

**Table 2:** Variable descriptive statistics, reliability and convergent validity

Construct	M	SD	Loading	CA	CR	AVE
Anthropomorphism (ANT) (Bartneck et al. 2009)				0.856	0.853	0.596
ANT1 *1 Fake – 5 Natural*	1.647	0.975	0.651			
ANT2 *1 Machinelike – 5 Humanlike*	1.633	1.067	0.686			
ANT3 *1 Unconscious – 5 Conscious*	1.841	1.084	0.829			
ANT4 *1 Artificial – 5 Lifelike*	1.994	1.175	0.897			
Hedonic Perceptions (HP) (Gursoy et al. 2019)				0.907	0.907	0.830
HP1 *Interacting with AI-powered service robots can be fun*	3.670	1.146	0.897			
HP3 *Interacting with a robot with artificial intelligence can be pleasant*	3.478	1.222	0.925			
Interaction Quality Perception (IQ) (Choi et al. 2020)				0.894	0.895	0.740
IQ1 *Human staff and service robots seem to give customers sympathetic, caring attention*	3.776	1.101	0.916			
IQ2 *Human staff and service robots seem to make customers feel comfortable*	3.760	1.107	0.828			
IQ3 *Human staff and service robots seem to communicate with customers smoothly*	3.539	1.189	0.833			
Perceived Safety (PS) (Jang and Lee 2020)				0.931	0.932	0.820
PS1 *Service robots are safe to use*	3.110	1.328	0.797			
PS2 *Service robots look safe*	3.396	1.218	0.966			
PS3 *Customer services are safe with robots in a service environment*	3.265	1.239	0.945			
Objections to use (OU) (Gursoy et al. 2019)				0.726	0.777	0.649
OU2 *I prefer human contact in service transactions*	4.158	0.967	0.980			
OU3 *People need emotional exchanges during service transactions*	3.995	1.071	0.582			
Attitudes Towards Service Robots (ATR) (Go and Sundar 2019)				0.802	0.806	0.587
ATR1 *Knowledgeable*	2.967	1.157	0.597			
ATR2 *Competent*	3.441	1.132	0.906			
ATR3 *Informed*	3.508	1.180	0.764			
Intention to recommend (IR) (Jung et al. 2015)				0.876	0.877	0.703
IR1 *I will recommend this AI device service to my friends and relatives*	3.214	1.120	0.869			
IR2 *When I return home, I will positively promote this AI device service*	3.280	1.056	0.801			
IR3 *I will strongly recommend others to use this AI device service*	3.028	1.124	0.845			
Willingness to accept Service Robots use (W) (Gursoy et al. 2019)				0.874	0.875	0.701
W1 *I am willing to receive robot services in restaurants*	3.584	1.188	0.884			
W2 *I would feel happy to interact with service robots in restaurants*	3.483	1.211	0.871			
W3 *I am likely to interact with service robots in restaurants*	3.559	1.249	0.750			

Note. M = Mean; SD = Standard deviation; CA = Cronbach’s alpha; CR = Composite reliability; AVE = Average variance extracted

### Structural model assessment

Through the evaluation of the structural model, an analysis was made of the significance of the hypothesised relationships and the predictive relevance of the proposed model. First, a bootstrapping procedure with 5000 subsamples was carried out to evaluate the significance of the trajectories of the coefficients (Hair et al. 2011). As can be seen in Table 4, all the model’s hypotheses received empirical support, apart from H6, H8 and H12.

Table 5 shows the predictive capacity values of the model. Specifically, the R^2^ values for all variables exceed the minimum limit of 0.1 (Falk and Miller 1992). The model explains much of the variance of the endogenous latent variables, willingness to accept service robots (73.6%) and intention to recommend (32.3%). In addition, the model also explains the variance of the constructs interaction quality perception (40.5%), attitudes towards service robots (34.7%) and objections to use (11.5%). The predictive capacities of the dependent constructs and the endogenous variables were also measured using the Q^2^ test and a blindfolding procedure (omission distance = 7) (Geisser 1975; Stone 1974). All results were greater than 0 (ranging from 0.062 to 0.458), so the proposed model has predictive relevance. In addition, the standardised root mean square residual (SRMR) was calculated as 0.039, lower than the acceptable maximum value of 0.08. The Normed Fit Index (NFI = 0.916) was higher than the 0.9 value considered to be the threshold. Thus, we can conclude that the model has goodness of fit.

In terms of direct effects, the construct willingness to accept service robots is mainly explained by the positive influence of intention to recommend (β15 = 0.781, p < 0. 001) and attitudes towards service robots (β11 = 0.093, p < 0.05); intention to recommend is mainly explained by attitudes towards service robots (β13 = 0.400, p < 0.001) and the negative influence of objections to use (β14 = – 0.299, p < 0.001); attitudes towards service robots are explained by hedonic perceptions (β3 = 0.293, p < 0.001), perceived safety (β8 = 0.178, p < 0.001), interaction quality perceptions (β5 = 0.134, p < 0.05), anthropomorphism (β1 = 0.117, p < 0.001) and by the negative influence of objections to use (β10 =– 0.139, p < 0.001); objections to use are explained mainly, and negatively, by anthropomorphism (β2 = − 0. 203, p < 0.001) and by hedonic perceptions (β4 =  − 0. 154, p < 0.001); finally, interaction quality is explained by perceived safety (β7 = 0.637, p < 0.001).

## Discussion and conclusions

### Theoretical implications

The present study offers important contributions to the body of knowledge about consumers’ willingness to use AI-powered service robots in restaurants and contributes to the literature by validating a model that verifies the applicability of BRT to explain intention to use service robots (as other authors have done in previous studies into other AI-based technologies) (e.g. Gesk and Leyer 2022; Lalicic and Weismayer 2021; Lin et al. 2022).

Specifically, the results provide a better understanding of the role of attitudes towards use, and objections to use, in the success of the implementation of service robots. The present study is original in proposing and empirically validating a behavioural model, based on reviews posted by customers who have experienced service robots in restaurants, that includes the specific attributes of the robots, and response variables such as consumers’ intention to recommend them and accept their use in restaurants.

Second, this study increases knowledge of the positive effects of four important attributes (reasons for) of the service robots used in restaurants on consumers’ attitudes, and their negative effects on objections (reasons against). Specifically, and in order of importance, hedonic perceptions, perceived safety, interaction quality perception and anthropomorphism were shown to have significant influence on attitudes towards use. This is an important contribution because no previous work on service robots in restaurants has evaluated these four antecedents of attitude in the same behavioural model (e.g. Chuah et al. 2022). In addition, the results showed that anthropomorphism and hedonic perceptions, in that order, contribute to reducing customer objections towards the use of service robots, while perceived safety and interaction quality perceptions had no significant effect. This contribution is novel because no previous work has identified these direct relationships, rather they have been shown to be mediated by other factors, such as performance expectancy, effort expectancy and positive emotions (e.g. Gursoy et al., 2019; Lin et al. 2020). It was also observed that, while hedonic perceptions is the antecedent with the greatest impact on attitude, anthropomorphism has the greatest impact on objections. Therefore, the findings contribute to the literature by identifying significant relationships and by allowing a comparison to be made of the effects of these four important antecedents of attitude, and objections towards the use of service robots in restaurants.

As previously commented, the results indicated that hedonic perceptions of the service performed by service robots have a positive effect on attitudes towards use and a negative effect on objections to use. This finding demonstrates that hedonic perceptions are the most important determining factor in customers' positive attitude, which is consistent with the arguments of Wei et al. (2016), Gursoy et al. (2019) and Vimalkumar et al. (2021). That is, consumers consider service robots as more hedonic than utilitarian, hence intrinsic motivation drives user acceptance (Lu et al. 2019). This result suggests that, currently, consumers regard their interactions with service robots more as fun, entertainment or an attractive technological novelty, than as a means of receiving a service equal or superior to that provided by humans. This result is important because, as Yuan et al. (2022) argued, it is necessary to understand how users perceive AI-powered devices, and their hedonic and utilitarian benefits, to promote their use. In this sense, at the present moment, the service robots employed in restaurants provide very limited services, so it is unsurprising that clients’ greatest motivation for accepting them is the pleasure and entertainment they derive from their interactions with the robots.

The results also indicated that perceived safety has a positive effect on attitudes towards use, while no significant relationship was found between perceived safety and objections to use. This provides support to the argument that the attributes of service robots positively affect consumers’ satisfaction with them (Jia et al. 2021), and their attitudes towards their use (Jang and Lee 2020). However, although perceived safety seems to improve attitude, it does not significantly reduce objections to the use of AI, as reluctance associated with job losses (Belanche et al. 2021c), privacy risks (Chuah et al. 2021) and service failures (Tussyadiah et al. 2020) remains. This is an important contribution as no previous study has evaluated the impact of perceived safety on objections. In addition, perceived safety was shown to have a significant and positive relationship with interaction quality perception. This result is in line with previous literature which also proposed that, as perceptions of safety increase, so do perceptions of service, this relationship being key at the time of the interaction, or the "moment of truth", when the service is delivered (Bartneck et al. 2009; Kandampully et al. 2018).

It was also shown that interaction quality perception had a positive effect on attitudes towards the use of service robots, while its relationship with objections to use was not significant. That is, when consumers perceive they can enjoy a quality interaction they develop a more positive attitude towards service robots, but this perception does not reduce the objections to use that arise based on the factors discussed above. This result supports the proposal that positive interactions with service robots encourage customers to use them (Chi et al. 2020).

Our results suggest that anthropomorphism positively affects attitudes towards use and decreases objections to use. Previous studies have returned conflicting results about the effect of anthropomorphism on user behaviour (Lv et al. 2022). While some works have shown that the anthropomorphic characteristics of AI-powered service robots positively influence variables such as engagement, social presence, usefulness, trust, attitudes and enjoyment (Li and Sung 2021; Li and Wang 2021), others have suggested that an excess of anthropomorphic characteristics can provoke rejection, or fear, in users, in line with uncanny valley theory (Martin et al. 2020). In this regard, it should be noted that the service robots used in the stimuli (i.e. videos) shown to the participants in the present study had mid-level anthropomorphic characteristics (see Web Appendix B). This allowed us to demonstrate the positive influence of this factor on intention to use. Therefore, it is reasonable to conclude that customers’ perceptions of the anthropomorphism of AI-powered service robots will provoke positive attitudes towards use, and decrease objections to use, at least for those with mid-level anthropomorphism. This is an important contribution to the literature because, while the anthropomorphism–attitude relationship has been analysed in previous studies, the relationship between anthropomorphism and objections has scarcely been examined.

Third, the results showed that objections to the use of service robots in restaurants negatively affect the customer's attitude towards their use. This result is novel as previous studies that incorporated objections into models of consumer behaviour regarded them as an outcome reflecting customers’ attitudes (e.g. Chi et al. 2022). In addition, it was shown that attitudes towards use, and objections to use (negatively), influenced intention to recommend. As for the effect of attitudes towards use on intention to recommend and willingness to accept, the effect of attitudes on behavioural intentions is widely accepted in the technology field (Oliver 2014) and, specifically, in the context of AI-powered devices (e.g. Mishra et al. 2021; Shin and Jeong 2020). Meanwhile, the finding of this study that objections to use exert a negative effect on intention to recommend can be considered reasonable, as these objections are based on the belief that the services provided by robots and other AI-powered devices cannot match those delivered by humans (Choi et al. 2020; Prentice et al. 2020), that they do not offer the social interaction demanded by users (Gursoy et al. 2019), nor address consumers’ concerns about risks to their personal information security/privacy. However, the results showed that the effect of objections on willingness to accept service robots, although negative, is not significant. Future studies should further analyse this relationship.

Fourth, the results of the study showed that intention to recommend has a strong influence on willingness to accept use, demonstrating that customers' acceptance of AI robotic devices is driven first and foremost by intention to recommend, that is, by their likelihood of recommending the restaurant or service to others in the future (Ryu et al. 2008); and second, by positive attitudes towards use (Gursoy et al. 2019).

### Managerial implications

New technologies are being continually introduced in all sectors to improve services and maintain customer satisfaction and pleasure (Tai et al. 2021). Many service companies are investing in the development and deployment of AI to increase their operational efficiency and to reduce costs. However, recent studies have suggested that not all customers are willing to accept AI devices to receive services (Gursoy et al. 2019; Lu et al. 2019). For this reason, companies must understand the willingness of customers to accept AI devices, in order not to waste resources, diminish the quality of services provided and even lose customers.

From a practical viewpoint, the consumer’s consumption experience is based on his/her cognitive and hedonic needs, with cognitive needs being addressed by technological applications that offer efficient, accurate and stable services. However, hitherto, human interaction has been necessary to identify, respond to, and satisfy, hedonic, or affective, needs (Tai et al. 2021).

The positive effects that hedonic perceptions have on attitudes and objections to use indicate that this attribute has the greatest effect on consumer opinion. Businesses can benefit from the entertainment and the novelty offered by AI devices, so they should emphasise these features to encourage customers to visit their establishments and experience interactions with service robots. That is, restaurant managers should take advantage of the hedonic appeal/benefits of the experience to attract customers. Going beyond the novelty effect, however, restaurants and technology companies should enhance their customers’ perceptions of the utility benefits they derive from using service robots. In this sense, restaurant managers might improve their customers’ service experiences by reinforcing the attributes of their service robots or, at least, making them appear more human and intelligent, by taking measures such as incorporating body language features. Advances in AI will enhance the capabilities of service robots by adding more empathetic and human aspects, thus providing better personal and autonomous care (Chiang and Trimi 2020). Although some works (see Belanche et al., 2020a) have indicated that customers prefer to be served by human waiters than by robots, the results of the present study show a good level of acceptance of their use that should encourage entrepreneurs in the sector to incorporate them not only as a lure, or attraction, but also as a means of addressing the lack of waiters suffered by countries with high tourist demand, such as Spain (Hosteltur 2022).

The study also demonstrated the important influence of perceived safety for consumers’ assessment of the quality of customer-service robot interactions. Therefore, before they introduce AI-powered robots, companies should ensure they meet or exceed customers’ expectations regarding interactions and safety. To this end, companies should go through transition periods in their introduction of service robots to evaluate consumer acceptance and, thus, minimise any negative effects.

### Limitations and future research

This study has limitations that open avenues to future work. First, the content analysis was performed on reviews only of one restaurant. Although, when the data were collected, the restaurant was a pioneer in Spain in the incorporation of service robots, other restaurants have since copied the strategy. Therefore, future work might use reviews of more restaurants to identify the reasons for, and against, the use of service robots. Second, the data come from a cross-sectional survey, so future studies might examine the stability of the relationships analysed using longitudinal data. Third, non-probabilistic sampling was employed, which can introduce bias into results. Therefore, future studies might use other sampling procedures, for example, by collaborating with restaurants that use AI-powered service robots. Fourth, the data were collected in Spain. Future studies might evaluate the model in different cultural contexts. Fifth, this study gathered data by collecting the responses of participants who viewed videos featuring service robots operating in restaurants. Future works might evaluate the validity of the model by using data from consumers who have actually experienced service robots in restaurants. Sixth, our results showed that objections to the use of service robots negatively influence consumers’ intentions to use the devices. Future studies might further analyse the impact of specific aspects that act as brakes to consumers’ willingness to accept service robots, such as their concerns about job losses, reduced social interaction or perceptions that service quality will be diminished. Similarly, while in the present study it was shown that anthropomorphism reduces objections, and positively influences attitude, no evidence was found as to whether these relationships vary based on the level of anthropomorphism of the robots. Therefore, future work might explore the strength of that effect.

**Table 3:** Discriminant validity

	ANT	ATR	HP	IQ	IR	OU	PS	W
Anthropomorphism (ANT)	*0.772*	0.180	0.057	0.146	0.322	0.205	0.033	0.178
Attitudes Towards Service Robots (ATR)	0.165	*0.767*	0.490	0.455	0.497	0.329	0.431	0.488
Hedonic Perceptions (HP)	0.011	0.494	*0.911*	0.552	0.527	0.260	0.522	0.716
Interaction Quality Perceptions (IQ)	0.148	0.459	0.552	*0.860*	0.619	0.235	0.637	0.672
Intention to recommend (IR)	0.319	0.491	0.528	0.619	*0.839*	0.439	0.610	0.851
Objections to use (OU)	− 0.210	− 0.304	− 0.241	− 0.237	− 0.421	*0.807*	0.210	0.436
Perceived Safety (PS)	− 0.026	0.443	0.522	0.637	0.611	− 0.214	*0.905*	0.657
Willingness to accept Service Robots (W)	0.179	0.495	0.714	0.671	0.852	− 0.418	0.657	*0.837*

The square roots of the AVEs are in italics on the main diagonal. The Fornell-Larcker criterion is depicted below the main diagonal. The heterotrait-monotrait (HTMT) ratio of correlations is above the main diagonal

**Table 4:** Results of the hypotheses testing

Hypothesis	Path coefficient	*t*-value	*p*-value*	Supported
H1. Anthropomorphism → Objections to use Service Robots	− 0.203	4.415	0.000	Yes
H2. Anthropomorphism → Attitudes Towards Service Robots	0.117	2.876	0.000	Yes
H3. Hedonic Perceptions → Objections to use Service Robots	− 0.154	2.922	0.004	Yes
H4. Hedonic Perceptions → Attitudes Towards Service Robots	0.293	5.071	0.000	Yes
H5. Perceived Safety → Interaction Quality Perceptions	0.637	21.139	0.000	Yes
H6. Perceived Safety → Objections to use Service Robots	− 0.104	1.599	0.110	No
H7. Perceived Safety → Attitudes Towards Service Robots	0.178	2.976	0.000	Yes
H8. Interaction Quality →Perceptions Objections to use Service Robots	− 0.056	0.897	0.370	No
H9. Interaction Quality →Perceptions Attitudes Towards Service Robots	0.134	1.991	0.047	Yes
H10. Objections to use Service Robots → Attitudes Towards Service Robots	− 0.139	3.022	0.003	Yes
H11. Objections to use Service Robots → Intention to recommend Service Robots	− 0.299	6.837	0.000	Yes
H12. Objections to use Service Robots → Willingness to accept Service Robots	− 0.061	1.870	0.062	No
H13. Attitudes Towards Service Robots → Intention to recommend Service Robots	0.400	8.768	0.000	Yes
H14. Attitudes Towards Service Robots → Willingness to accept Service Robots	0.093	2.28	0.023	Yes
H15. Intention to recommend → Willingness to accept Service Robots	0.781	24.636	0.000	Yes

*n* =5000 subsamples. *95% confidence level – two tailed

**Table 5:** Assessment of the structural model

Constructs	R^2^	Q^2^	β	Correlations	Explained Variance
*Attitudes Towards Service Robots*	0.347	0.188			
Anthropomorphism			0.117***	0.165	0.019
Hedonic Perceptions			0.293***	0.494	0.145
Perceived Safety			0.178***	0.443	0.079
Interaction Quality Perceptions			0.134*	0.459	0.062
Objections to use Service Robots			− 0.139***	− 0.304	0.042
*Objections to use Service Robots*	0.115	0.062			0.000
Anthropomorphism			- 0.203***	− 0.210	0.043
Hedonic Perceptions			- 0.154***	− 0.241	0.037
Perceived Safety			- 0.194^ ns^	− 0.214	0.042
Interaction Quality Perceptions			- 0.056^ ns^	− 0.237	0.013
*Interaction Quality Perceptions*	0.405	0.277			0.000
Perceived Safety			0.637***	0.637	0.406
*Intention to recommend Service Robots*	0.323	0.197			0.000
Objections to use Service Robots			- 0.299***	− 0.421	0.126
Attitudes Towards Service Robots			0.400***	0.491	0.196
*Willingness to accept Service Robots*	0.736	0.458			0.000
Objections to use Service Robots			- 0.061^ ns^	− 0.418	0.025
Attitudes Towards Service Robots			0.093**	0.495	0.046
Intention to recommend Service Robots			0.781***	0.852	0.665

*ns* = not significant.

***p < 0.001; **p < 0.01; *p < 0.05

## Supplementary Information

Below is the link to the electronic supplementary material.

Supplementary file1 (DOCX 328 KB)
