# Supplementary material for: Exploring the antecedents of customers’ willingness to use service robots in restaurants
Source: Serv Bus. 2022 Nov 2;17(1):167–93. doi: 10.1007/s11628-022-00509-5 (PMC9628411; doi:10.1007/s11628-022-00509-5)
Supplement: Supplementary file 1 — Supplementary file1 (DOCX 328 KB) [file 11628_2022_509_MOESM1_ESM.docx]

**WEB APPENDIX A.** A non-exhaustive list of recent relevant articles on service robot adoption in the hotel and restaurant industries (sorted alphabetically by author)

| Source | Theory | Methodology | Aim | Field | Predictors (P) and outcomes (O) | Findings |
| --- | --- | --- | --- | --- | --- | --- |
| Belanche et al. (2020c) | Attribution theory. | Two experimental studies based on vignettes. | “To compare customer attributions in service encounters where the agent is a human (employee) to those in encounters where the agent is a frontline robot” (p. 268). | Hotels and restaurants. | P: frontline agent (robot vs human), service outcome (failure vs success).  O: Agent´s responsibility, firm’s responsibility, stability. | Users “make stronger attributions of responsibility for service performance towards humans than towards robots” (p. 267). “Customers expect employees to improve after a poor service encounter but expect little improvement in robots’ performance over time” (p. 267). |
| Belanche et al. (2021c) | Attribution theory and literature on customers’ perceptions about robots. | Survey based on pictures and descriptions. | “To explore how customers’ attributions about firms’ motivations to introduce service robots affect their intentions to use and recommend” (p. 477). | Restaurants. | P: Human-likeness, perceived affinity, service enhancement, cost reduction.  O: Intention to use and intention to recommend. | “Attributions mediate the relationships between affinity towards robots and customer behavioural intentions to use and recommend service robots” (p. 477). |
| Cha (2020) | Cognitive evaluation theory and the theory of planned behaviour. | Survey based on a video and snapshots. | “Defining customers’ intentions to use the services of restaurant robots in Korea” (p. 2947). | Restaurants. | P: coolness, motivated consumer innovativeness, attitude, perceived value.  O: Intention to use. | “Hedonically motivated consumer innovativeness and socially motivated consumer innovativeness have positive effects on attitude and are enhanced by attractiveness, utility, subcultural appeal and originality” (p. 2947). |
| Chan and Tung (2019) | Brand experience literature. | Two experiments: service delivery (human or robot); service delivery: 2 (human or robot) x 3 (hotel segment). | “Effects of robotic service on guests’ evaluations of hotel brand experience” compared to service delivery by human staff, and “the moderating effects of hotel segment” (p. 458). | Hotel industry. | P: Who offers the service (human or robot) and hotel segment.  O: brand experience (sensory, affective, behavioural, and intellectual). | The use of service robots “may not necessarily enhance brand experience. Their effects were seen to be moderated by hotel type; brand experience was enhanced in budget and midscale hotels, but not in luxury hotels” (p. 458)). |
| Chiang and Trimi (2020) | Expectation disconfirmation theory and SERVQUAL. | Importance performance analysis (IPA) and the Technique for Order Preference by Similarity to an Ideal Solution (TOPSIS). | “To explore the service quality provided by robots based on real data in a hotel setting” (p. 439). | Hotel industry. | P: Customer inputs, technical development of service robots.  O: Service quality of service robots and their importance ranking. | “Customers’ top priorities for robots’ service quality are assurance and reliability, while tangible and empathy were not as important” (p. 439). |
| Choi et al. (2020) | Service quality models (e.g., SERVPERF, SERVQUAL) and computers are social actors” (CASA). | Focus-group interviews with hotel managers.  Experiment to compare hotel guests’ perceptions about services. | “To understand hotel managers’ expectations of the future use of service robots and their effect on service quality, and to examine and compare hotel guests’ perceptions of service quality between service robots and human staff” (p. 615). | Hotel industry (including food-delivery service at restaurant). | 3 conditions: human staff only, service robot only, both human staff and service robot.  Variables to compare interaction quality, outcome quality and physical service environment. | “Human staff services are perceived as better than the services of service robots in terms of interaction quality and physical service environment. However, no significant difference in outcome quality was noted” (p. 613)". |
| Chuah et al. (2022) | Theory of consumption values. | Survey based on a video. | “To explore customer value perceptions of service robots and their impact on customers’ attitudes and behaviours towards robotic restaurants” (p. 49). | Restaurants. | P: Functional value, emotional value, social value, epistemic value, co-creation value, conditional value.  O: Willingness to use, willingness to pay more. | “Customers’ willingness to use and to pay more for robotic restaurants are determined by their attitudes towards robots, which are influenced by functional, conditional, epistemic, emotional, co-creation and social values” (p. 49). |
| Fan et al. (2020) | Service robot acceptance model, self-service technology (SST) research and anthropomorphism literature. | 2 (self-service machine type) by 2 (interdependent self-construal) by 2 (technology self-efficacy) between-subjects experiment. | To study “the possible influence of machine anthropomorphism on consumer blame attributions and dissatisfaction after experiencing a service failure” (p. 270). | Hotel industry. | P: Technology anthropomorphism, technology self-efficacy, interdependent self-construal, blame attribution.  O: Consumer dissatisfaction. | Consumers show “different levels of dissatisfaction with a service failure caused by an anthropomorphic self-service machine based on their levels of interdependent self-construal and technology self-efficacy” (p. 281). |
| Fuentes-Moraleda et al. (2020) | Service robot acceptance model (sRAM). | Content analysis of online TripAdvisor reviews. | To analyse “the Service Robot Acceptance Model (sRAM) from the perspective of human-robot interactions in hotels that have introduced service robots” (p. 2). | Hotel industry. | - | “Hotel guests interact with robots predominantly through the functions the robots perform; reception, room service and baggage handling” (p. 9). |
| Fusté-Forné (2021) | Grounded theory approach. | Semi structured interviews with tourists. | To investigate “tourists’ perceptions of the use of robots in restaurants” (p. 1). | Restaurants. | - | “Identify advantages and disadvantages. Tourists perceive robots in food services as a form of dehumanisation of the gastronomic experience” (p. 1). |
| Guan et al. (2021) | Stimulus-organism-response (SOR) theory and the servicescape model. | Questionnaire survey of robot using restaurant customers. | “To explore the impact of the robot restaurant servicescape and robot service competence (RSC) on customers’ behavioural intentions, and to analyse the mediating role of hedonic value (HV) and utilitarian value (UV)” (p. 1). | Restaurants. | P: servicescape, robot service competence, negative attitude towards robots, openness to change, hedonic value, utilitarian value.  O: Behavioural intention. | Servicescape and RSC improve customers’ behavioural intentions. In addition, HV and UV mediate the influence of servicescape and RSC on customer behavioural intention. OC negatively moderates the influence of servicescape on UV, and negative attitude towards robots (NAR) negatively moderates the influence of RSC on HV (p. 1). |
| Ivanov et al. (2020) | Technology-organisation-environment framework, UTAUT and Institutional theory. | Structured quantitative data and qualitative data from managers. | “To evaluate the perceptions of Bulgarian hotel managers of service robots” (p. 508). | Hotel industry. | Advantages, disadvantages, experience. | “Repetitive, dirty, dull and dangerous tasks in hotels would be more appropriate for robots, while hotel managers would rather use employees for tasks that require social skills and emotional intelligence” (p. 527). |
| Kervenoael et al. (2020) | Human robot interaction (HRI) literature and TAM. | Interviews and on-site survey. | “To bring a clearer understanding of the dynamics that prevail in visitors' intention to use social robots in the context of robotised hospitality services” (p. 2). | Hospitality services. | P: Personal engagement, service assurance, empathy, perceived ease of use, perceived usefulness, perceived value, information sharing, tangibles.  O: Intention to use. | “Visitors' intentions to use social robots stem from the effects of technology acceptance variables, service quality dimensions leading to perceived value, and two further dimensions from human robot interaction (HRI): empathy and information sharing” (p. 1). |
| Kuo et al. (2017) | Six-dimensional service innovation model. | Interviews (hospitality and robotics experts). | “Identify the factors that influence the development of service robots, and to apply a service innovation strategic mindset to the hotel industry” (p. 1305). | Hotel industry. | - | “Service robots can help hotels handle seasonal employment and labour utilisation” (p. 1305). |
| Lin et al. (2020) | Artificially intelligent device use acceptance (AIDUA) model. | Survey using an online customer panel. | “To validate and extend the applicability of the AIDUA model for explaining customers’ AI service device acceptance in the hospitality service context” (p. 532). | Hotel industry. | P: Social influence, hedonic motivation, anthropomorphism, performance expectancy, effort expectancy, positive emotion.  O: Willingness to use AI devices, objection to the use of AI devices. | Validation of the “applicability of the AIDUA framework, suggesting that hospitality customers’ intentions to use AI devices are influenced by social influence, hedonic motivation, anthropomorphism, performance and effort expectancy, and emotions felt towards the devices” (p. 530). |
| Lu et al. (2019) | Unified theory of acceptance and use of technology 2 (UTAUT2) | Psychometric procedure for scale development. | Development and validation of a multidimensional scale for the evaluation of the predisposition towards the integration of service robots (Service Robot Integration Willingness-SRIW). | Service sectors (including restaurants). | P: performance efficacy, intrinsic motivation, anthropomorphism, social influence, facilitating conditions, emotions.  O: willingness to use service robots. | Identification of six dimensions: five positive (performance efficacy, intrinsic motivation, social influence, facilitating conditions, emotions) and one negative (anthropomorphism).  Hedonic dimensions (intrinsic motivation, emotions) influence adoption. |
| Lu et al. (2021) | Appraisal theory. | 3 (appearance human-likeness) × 2 (voice human-likeness) × 2 (language style human-likeness) between-subjects experiment using experimental vignette methodology. | “How service robots’ varying levels of human-likeness influence consumption outcomes, and the underlying mechanisms involved, through cognition and positive emotion, as per appraisal theory” (p. 1). | Restaurants. | P: Appearance human-likeness, voice human-likeness, language style human-likeness.  O: Service encounter evaluation, loyalty, WOM intentions, perceived credibility, emotion. | “Humanlike language style positively affects service encounter evaluation. The significant effect of a humanlike voice on three consumption outcomes is only explained by positive emotion, whereas the effect of humanlike language style on service encounter evaluation is explained by both emotion and cognition” (p. 1). |
| Shin and Jeong (2020) | Uncanny valley theory. | 3 × 2 × 3 between-subjects factorial design experiment. | “To examine the effects of robot concierges on hotel guests’ attitudes and adoption intentions towards robot concierges” (p. 2613). | Hotel industry. | P: Morphology of robot concierges, level of interactivity, level of hotel service, attitude towards robot concierges.  O: Adoption intention. | “The robot’s morphology significantly influenced guests’ attitudes towards robot concierges. Even if guests had favourable attitudes towards robot concierges, they preferred human employees” (p. 2613). |
| Tussyadiah and Park (2018) | Human robot interaction (HRI) literature. | Online survey and laboratory experiment. | “To understand consumer evaluation of hotel service robots and its effects on adoption intention, and to identify if consumers react differently to different types of hotel service in light of their operational capabilities” (p. 309). | Hotel industry. | P: Anthropomorphism, animacy, likeability, perceived intelligence, perceived security, importance of operations.  O: Adoption intention. | “Adoption of hotel service robots is significantly influenced by dimensions of HRI: anthropomorphism, perceived intelligence, and perceived safety” (p. 318). |
| Xu et al. (2020) | Organisational change theory. | Three-stage Delphi study with hotel industry human resource experts. | “To identify the key trends and major challenges that will emerge in the next ten years and assess how business leaders might deal with the challenges brought about by service robot technologies” (p. 2217). | Hotel industry. | - | Service robots “increase the efficiency and productivity of hotel activities, but also pose challenges, such as their high cost, skills deficits and the significant changes they involve to the organisational structure and culture of hotels” (p. 2217). |
| Zhong et al. (2020) | Theory of planned behaviour, technology acceptance model and the perceived value-based acceptance model. | Online and on-site surveys. | “To study customers' recognition and acceptance of hotel service robots to guide the successful promotion of this technology” (p. 1325). | Hotel industry. | P: Sentimental value, perceived behavioural control, usefulness, ease of use, self-efficacy, attitude, experience of hotel robot use.  O: Behavioural intention. | “Attitude, usefulness and perceived value are the factors that have the greatest impact on acceptance” (p. 1325). |

**WEB APPENDIX B.** Stimuli (video frames)


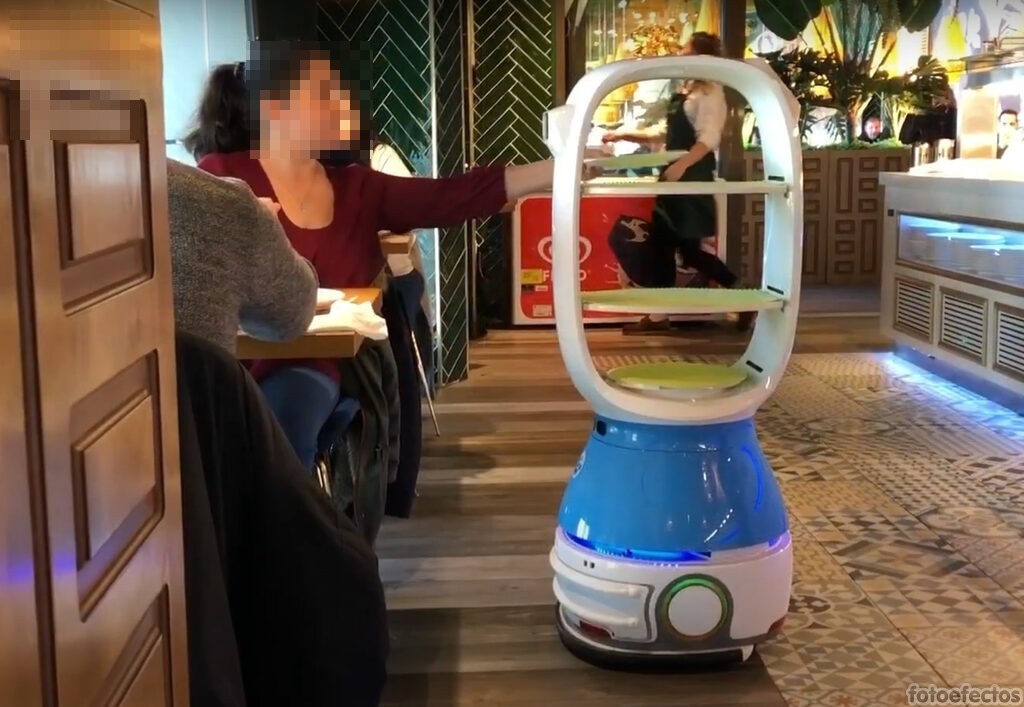

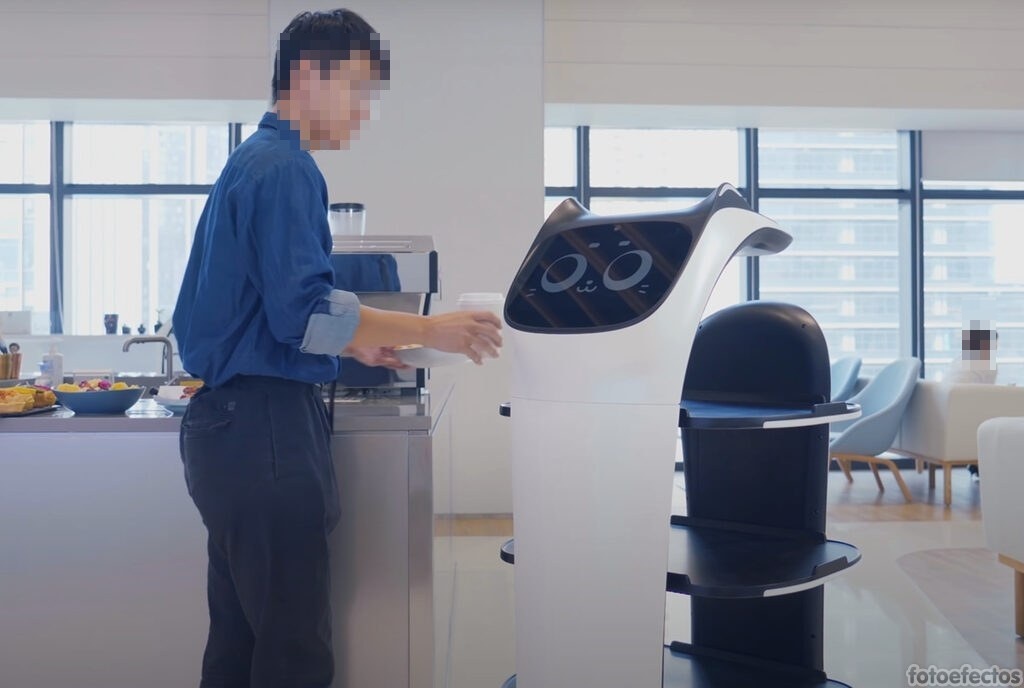


## References

Belanche D, Casaló LV, Flavián C, Schepers J (2020c) Robots or frontline employees? Exploring customers’ attributions of responsibility and stability after service failure or success. J Serv Manage 31(2):267-289

Belanche D, Casaló LV, Flavián C (2021c) Frontline robots in tourism and hospitality: service enhancement or cost reduction? Electron Markets 31:477-492

Cha SS (2020) Customers’ intention to use robot-serviced restaurants in Korea: relationship of coolness and MCI factors. Int J Contemp Hosp Manag 32(9):2947-2968

Chan APH, Tung VWS (2019) Examining the effects of robotic service on brand experience: the moderating role of hotel segment. J Travel Tour Mark 36(4):458-468

Chiang AH, Trimi S (2020) Impacts of service robots on service quality. Serv Bus 14:439-459

Choi Y, Choi M, Oh M, Kim S (2020) Service robots in hotels: understanding the service quality perceptions of human-robot interaction. J Hosp Market Manag 29:613-635

Chuah SHW, Aw ECX, Cheng CF (2022) A silver lining in the COVID-19 cloud: examining customers’ value perceptions, willingness to use and pay more for robotic restaurants. J Hosp Market Manag 31(1):49-76

Fan A, Wu L, Miao L, Mattila AS (2020) When does technology anthropomorphism help alleviate customer dissatisfaction after a service failure? - The moderating role of consumer technology self-efficacy and interdependent self-construal. J Hosp Market Manag 29:269-290

Fuentes-Moraleda L, Díaz-Pérez P, Orea-Giner A, Muñoz- Mazón A, Villacé-Molinero T (2020) Interaction between hotel service robots and humans: a hotel-specific Service Robot Acceptance Model (sRAM). Tour Manag Perspect 36:100751. <https://doi.org/10.1016/j.tmp.2020.100751>

Fusté-Forné F (2021) Robot chefs in gastronomy tourism: what's on the menu? Tour Manag Perspect 37:100774. <https://doi.org/10.1016/j.tmp.2020.100774>

Guan X, Gong J, Li M, Huan TC (2021) Exploring key factors influencing customer behavioral intention in robot restaurants. Int J Contemp Hosp Manag 34(9):3482-3501

Ivanov S, Seyitoğlu F, Markova M (2020) Hotel managers’ perceptions towards the use of robots: a mixed-methods approach. Inf Technol Tourism 22:505-553

Kervenoael RD, Hasan R, Schwob A, Goh E (2020) Leveraging human–robot interaction in hospitality services: incorporating the role of perceived value, empathy, and information sharing into visitors' intentions to use social robots. Tourism Manage 78:104042. <https://doi.org/10.1016/j.tourman.2019.104042>

Kuo C-M, Chen L-C, Tseng C-Y (2017) Investigating an innovative service with hospitality robots. Int J Contemp Hosp Manag 29(5):1305-1321

Lin H, Chi, O. H, Gursoy D (2020) Antecedents of customers’ acceptance of artificially intelligent robotic device use in hospitality services. J Hosp Market Manag 29:530-549

Lu L, Cai R, Gursoy D (2019) Developing and validating a service robot integration willingness scale. Int J Hosp Manag 80:36-51

Lu L, Zhang P, Zhang TC (2021) Leveraging “human-likeness” of robotic service at restaurants. Int J Hosp Manag 94:102823. <https://doi.org/10.1016/j.ijhm.2020.102823>

Shin HH, Jeong M (2020) Guests’ perceptions of robot concierge and their adoption intentions. Int J Contemp Hosp Manag 32(8):2613-2633

Tussyadiah IP, Park S (2018) Consumer evaluation of hotel service robots. In: Stangl B., Pesonen J. (eds) Information and Communication Technologies in Tourism 2018. Springer, Cham. <https://doi.org/10.1007/978-3-319-72923-7_24>

Xu S, Stienmetz J, Ashton M (2020) How will service robots redefine leadership in hotel management? A Delphi approach. Int J Contemp Hosp Manag 32(6):2217-2237

Zhong L, Zhang X, Rong J, Chan HK, Xiao J, Kong H. (2020) Construction and empirical research on acceptance model of service robots applied in hotel industry. Ind Manag Data Syst 121:1325-1352
